# Prevalence, patterns of multimorbidity and associations with health care utilization among middle-aged and older people in China

**DOI:** 10.1186/s12889-023-15412-5

**Published:** 2023-03-21

**Authors:** Yaqin Zhong, Gang Qin, Hanqing Xi, Duanying Cai, Yanan Wang, Tiantian Wang, Yuexia Gao

**Affiliations:** 1 School of Public Health, Nantong University, 9 Se-yuan Road, Nantong, Jiangsu 210029 China; 2 Clinical Trial Center, Affiliated Hospital of Nantong University, 20 Xi-Si Road, Nantong, Jiangsu 226001 China; 3 School of Medicine, Nantong University, 9 Qixiu Road, Nantong, Jiangsu 226019 China; 4 School of Nursing, Jiujiang University, 551 Qianjin Dong Road, Jiujiang, Jiangxi Province 332005 China

**Keywords:** Multimorbidity, Health care utilization, Chronic disease, China Health and Retirement Longitudinal Study

## Abstract

**Background:**

Multimorbidity has become one of the main challenges in health care system. The association between prevalence, patterns of multimorbidity and health care utilization is less often discussed in China. The purpose of this study is to examine this association among Chinese middle-aged and older adults and take into account different sociodemographic, behavioral and health characteristics. Based on this, implications of current evidence and effective intervention on multimorbidity and health care utilization can be identified and put into practice.

**Methods:**

The wave 4 in 2018 of the China Health and Retirement Longitudinal Study (CHARLS) was used in the study. Multimorbidity was defined as the co-occurrence of two or more chronic medical condition of a list of fourteen chronic diseases in one person. The presence of chronic diseases was assessed through self-report. Health care utilization include whether the respondents received outpatient service last month and inpatient service in the past year. Latent Class Analysis was conducted to identify the clustering pattern of chronic diseases. Logistic regressions were employed to explore the association between prevalence, patterns of multimorbidity and health care utilization. Analyses were weighted using individual sample weights, adjusted for non-response of individual and household.

**Results:**

Among 19,559 participants aged 45 and older, 23.10% were aged above 70 years and 52.42% were female. The prevalence of multimorbidity was 56.73%. Four patterns were identified: relatively healthy class, respiratory class, stomach-arthritis class and vascular class. Multimorbid individuals used more outpatient services (OR = 1.89, 95%CI = 1.65–2.17) and more inpatient services (OR = 2.52, 95%CI = 2.22–2.86) compared to their no-multimorbid counterparts. Compared to relatively healthy class, the respondents classified into respiratory class, stomach-arthritis class and vascular class used more outpatient services (OR = 1.90, 95%CI = 1.57–2.30; OR = 2.39, 95%CI = 2.06–2.78; OR = 1.53, 95%CI = 1.32–1.79 respectively) and more inpatient services (OR = 2.19, 95%CI = 1.83–2.62; OR = 2.93, 95%CI = 2.53–3.40; OR = 1.90, 95%CI = 1.65–2.19 respectively).

**Conclusion:**

Our study provided evidence that multimorbidity is high among Chinese older adults and is associated substantially higher health care utilization in China. Four multimorbidity patters were identified. Policy should prioritize improving the management of individuals with multimorbidity to increase healthcare efficiency. Further research is necessary with special emphasis on the trajectory of multimorbidity and the role of health system in satisfying needs of multimorbid individuals.

**Supplementary Information:**

The online version contains supplementary material available at 10.1186/s12889-023-15412-5.

## Background

Age-associated multiple chronic disease (multimorbidity) has been recognized as a critical public health issue around the world [1]. Multimorbidity poses a major challenge to health care symptoms, both in developing and developed countries [1, 2]. In geriatric individuals multimorbidity is associated with frailty, polypharmacy, health service misuse, with consequences of reduced quality of life, higher mortality and stress on health care systems [3–7]. Studies on multimorbidity are important for informing improvement of disease treatment, configuration of medical facilities and medical resource allocation [8].

Systematic reviews reported the prevalence of multimorbidity ranged from 3.5% (at age 75) to 100% (at age 85) around the world [1, 9]. The prevalence rises with increasing age and in the general population, an S-shape curve for prevalence by age was detected [10]. However, these results vary across studies due to the heterogeneity in the operational definition of multimorbidity, sample selection and methodology. Some articles examined prevalence for multimorbidity among Chinese adults, two of them were embedded in the same population-based study from the China Health and Retirement Longitudinal Study (CHARLS) [4, 11]. Yao [4] found multimorbidity occurred in 42.4% of participants aged at least 50 years and Chen [11] found the prevalence was 45.5% among urban residents. The prevalence among the whole sample and the association with health care utilization need further discussion.

A positive association between multimorbidity and health care utilization has been reported in some counties. Findings from Switzerland have showed that, the mean number of consultations was 15.7 among multimorbid individuals per year compared to 4.4 in the non-multimorbid sample [12]. Evidence from 16 European countries showed number of chronic diseases was associated with more hospital care utilization in both primary and secondary setting [13].

Previous literature has indicated that chronic conditions tend to cluster together into so-called multimorbidity patterns [14]. A study conducted in Brazil identified three patterns: cardiopulmonary pattern, musculoskeletal pattern and vascular-metabolic pattern among older adults [15]. Roselyne et al. found participants aged 60 years and older from the Swedish National Study and Care were grouped into six multimorbidity patterns: (i) psychiatric disorders (5.87%); (ii) cardiovascular diseases (6.27%); (iii) metabolic and sleep disorders (10.67%); (iv)sensory impairments and cancer (11.87%), (v) musculoskeletal, respiratory and gastrointestinal diseases (15.78%) and (vi) unspecific(49.56%) [14]. These findings may not be comparable due to differences in study population, analytical methods and eligible diseases. Recent studies have revealed the existence of multimorbidity patterns clustering systematically associated health problems that fall beyond the standard concept of medical specialties established by health systems. The disease cluster poses a challenge for the design of adequate prevention and treatment strategies [5].Some studies looked at multimorbidity patterns and hospital care utilization, but including only respondents aged 80 years or older, or already hospitalized patients [14, 16]. These associations were less discussed in China. Further research is needed in China.

The applicability of multimorbidity requires further knowledge of the prevalence, patterns, the chronic diseases that are involved, and the potential association with health care utilization. Research on prevalence and patterns of multimorbidity and the association with health care utilization is necessary among Chinese older adults, which can provide implications for clinical practice and health policy. The purpose of the present study is to assess multimorbidity and patterns of multimorbidity and explore the associations with health care utilization among middle-aged and older people in China. Based on this, implications of current evidence and effective intervention on multimorbidity and health care utilization can be identified and put into practice.

## Methods

### Data and sample

**Fig. 1:**
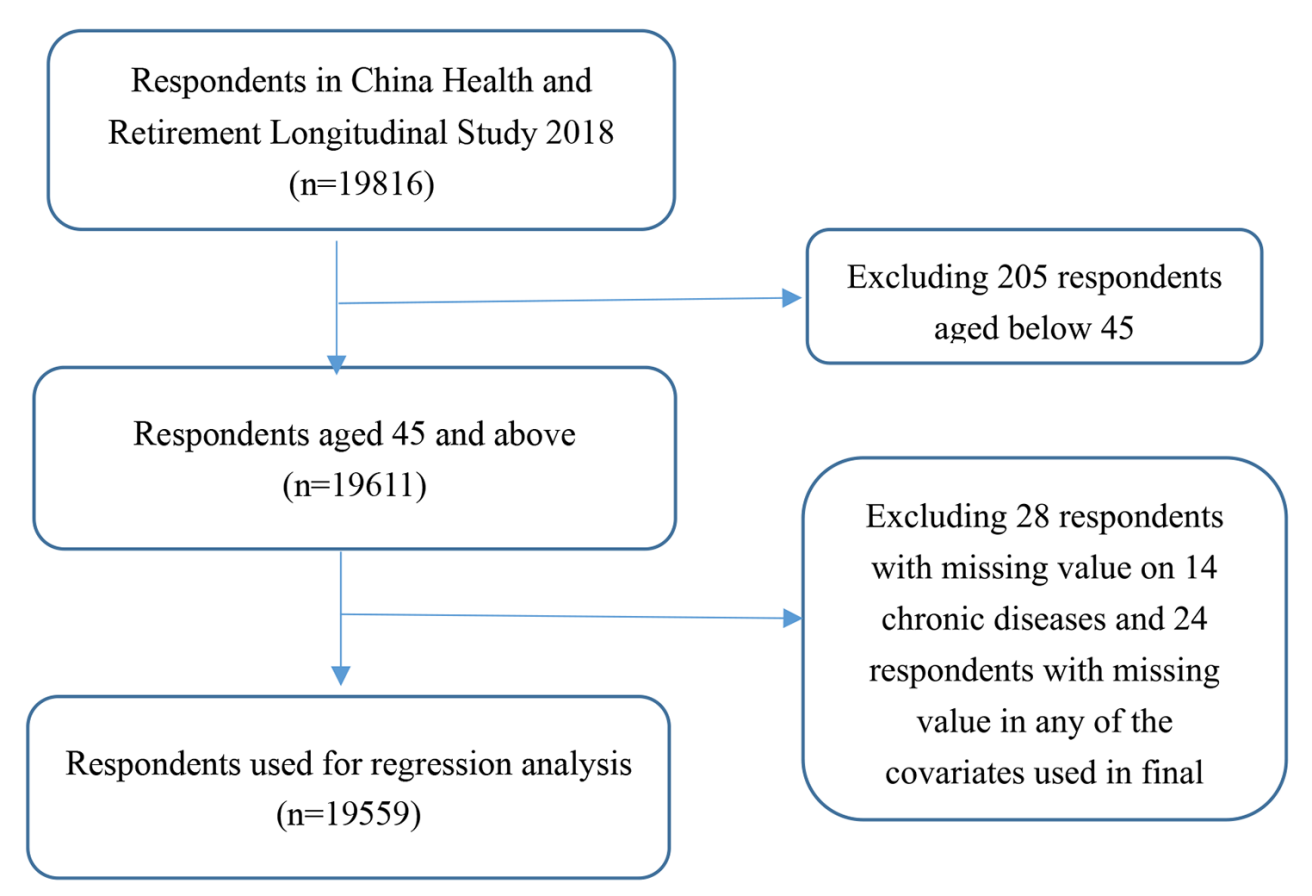
The flowchart of the sample

This study used data of wave 4 in 2018 of the China Health and Retirement Longitudinal Study (CHARLS). CHARLS is a nationally representative longitudinal survey of Chinese aged 45 and older. The CHARLS sample was obtained through stratified multistage probability proportional to size (PPS) sampling [17]. The CHARLS 2018 covered 150 counties/districts, 450 communities/villages, involving 19,816 individuals (overall response rate was 83.84%). The survey was conducted from June to August in 2018. Respondents who was under 45 years old and with missing values in chronic diseases and other important variables were exclude from analyses (n = 257). This study comprised 19,559 individuals aged 45 years old and above (Fig. 1).

### Ethics approval

The CHARLS was approved by the Biomedical Ethics Review Committee of Peking University (IRB00001052-11015) and all participants sighed informed consent before interview [17].

### Measures

#### Multimorbidity and multimorbidity patterns

Xu detected three major definitions of multimorbidity from three major perspectives: epidemiology and public health, clinicians and patients in daily clinical practice and long term care and family medicine in primary care [1]. The definition from WHO was the co-occurrence of two or more chronic medical condition in one person, which is simple and easy to implement. In this study, as most studies, multimorbidity was defined as the simultaneous presence of two or more chronic diseases/conditions [11]. In CHARLS, the respondents were asked whether they had been diagnosed by a doctor with fourteen chronic diseases including: hypertension, dyslipidemia, diabetes, cancer, chronic lung diseases, liver diseases, heart diseases, stroke, kidney diseases, digestive diseases, emotional or psychiatric problems, memory-related disease, arthritis and asthma. If the respondents answered “yes”, they were regarded as have the chronic diseases and the number of chronic diseases were computed. Consistent with previous literature [18], all 14 chronic diseases were included in the assessment of multimorbidity. Respondents with cognitive impairments were included in the study, if he/she cannot answer the question, his or her spouse will answer instead.

Previous studies showed that chronic diseases tend to cluster together into so-called multimorbidity patterns [14, 19]. Latent Class Analysis (LCA) was conducted to identify the clustering pattern of fourteen chronic diseases among the respondents. Four patterns were named as: relatively healthy class, respiratory class, stomach-arthritis class and vascular class (see Statistical Analysis section).

#### Health care utilization

Health care utilization include whether the respondents received outpatient service last month and inpatient service in the past year. If the respondents visited a public/private hospital, public health center, clinic, or health worker’s or doctor’s practice, or been visited by a doctor for outpatient care, the dummy variables of outpatient service utilization was coded as 1, otherwise as 0. Similarly, if the respondents received inpatient care in the past year, the dummy variable of inpatient service utilization was coded as 1, otherwise as 0.

#### Covariates

Guided by the literature [18, 20], covariates involved demographic characteristics, socioeconomic status, health behavior, health status and functional state. Demographic characteristics consisted of age group (45–59, 60–69, ≧70), gender (male, female), marriage status (married, windowed, others). Socioeconomic variables consisted of education (Sishu/home school and below, elementary school, middle school, high school and above), residence (rural, urban) and health insurance (UEBMI, URBMI, NRCMS, others, no insurance). We didn’t conclude work status because informal employment is an universal phenomenon in China. Many respondents in rural areas engage in agriculture work till they are very old. So we didn’t conclude this variable in the analysis. Health behavior included alcohol consumption (never, occasionally, usually) and smoking status (never, quit, current) [20]. Health status were measured by self-rated health (less than good, good). Functional state was measured by whether respondents have difficulty in activities in daily life (ADL, yes or no).

### Statistical analysis

LCA is a useful tool for determining subtypes or groups in multivariate categorical data. Two to six classes were examined and the best fitting solution was selected based on the evaluation of a variety of model fit statistics. The four-class model emerged as the best fitting one (see Supplementary Table 1 for the model-fit statistics) and had the most reasonable clinical results for interpretability. Four patterns were named as: relatively healthy class, respiratory class, stomach-arthritis class and vascular class. Descriptive statistics were conducted to explore the difference between socioeconomic, health related variables and multimorbidity characteristics. Logistic regression model was conducted to explore the association between multimorbidity and health care utilization. All models were adjusted for age, gender, marital status, education, residence, alcohol consumption, smoking, activities in daily life (ADL) and self-rated health (SRH). The analyses were weighted using individual sample weights, adjusted for non-response of individual and household. The level of significance was defined as 2-sided *P* value < 0.05. All analyses were conducted using Stata 13.0 (Stata Corp, College Station, TX, USA).

## Results

### Characteristics of the respondents

A total of 19,559 respondents aged 45 years old and above were included in the study. The socio-economic characteristics, multimorbidity factors and health behavior of all participants are shown in Table 1. In the sample, the proportion of the female (52.42%) was greater than the male (47.58%). 84.97% were married and 12.84% were windowed. For education, 43.65% had Sishu/home school and below. 59.69% lived in rural area. 26.13% of the respondents usually drank and 40.01% were currently smokers. 18.48% of the respondents had difficulty in ADL and 77.03% rated their health as less than good (Table 1).

**Table 1:** Sociodemographic factors, health behavior and morbidity characteristics of all respondents (n = 19,559)

Variables	Total (%)	Prevalence of multimorbidity	*P* value
**All participants**	19,559 (100%)	56.73%	
**Age**			
45–59	8504 (43.48%)	44.15%	< 0.001
60–69	6536 (33.42%)	62.86%	
≧70	4519 (23.10%)	69.42%	
**Gender**			
Male	9307 (47.58%)	54.65%	< 0.001
Female	10,252(52.42%)	58.71%	
**Marriage status**			
Married	16,619(84.97%)	55.20%	< 0.001
Windowed	2512 (12.84%)	66.58%	
Others	428 (2.19%)	53.37%	
**Education**			
Sishu/home school and below	8537 (43.65%)	60.49%	< 0.001
Elementary school	4289 (21.93%)	54.99%	
Middle school	4258 (21.77%)	53.37%	
High school and above	2475 (12.65%)	54.18%	
**Residence**			
Rural	11,675(59.69%)	56.21%	0.184
Urban	7884 (40.31%)	57.26%	
**Alcohol consumption**			
Never	12,899(65.95%)	60.44%	< 0.001
Occasionally	1466 (7.50%)	50.83%	
Usually	5111 (26.13%)	50.66%	
**Smoking status**			
Never	11,211(57.32%)	56.93%	< 0.001
Quit	445 (2.28%)	67.11%	
Current	7825 (40.01%)	56.55%	
**Difficulty in ADL**			
Yes	3615 (18.48%)	81.72%	< 0.001
No	15,944(81.52%)	51.25%	
**Self-rated health**			
Less than good	15,066(77.03%)	64.43%	< 0.001
Good	4493 (22.97%)	31.83%	
**Health insurance**			
None	583 (2.98%)	48.96%	< 0.001
UEBMI	2975 (15.21%)	60.63%	
URBMI	3189 (16.30%)	55.72%	
NRCMS	12,598 (64.41%)	56.20%	
Others	214 (1.09%)	49.79%	

ADL: Activities in Daily Life; UEBMI: Urban Employee Basic Medical Insurance; URBMI: Urban Resident Basic Medical Insurance; NRCMS: New Rural Cooperative Medical Scheme; Others: private medical insurance and other medical insurance

Prevalence of multimorbidity were weighed results adjusted for non-response of individual and household

### Prevalence and patterns of multimorbidity

**Fig. 2:**
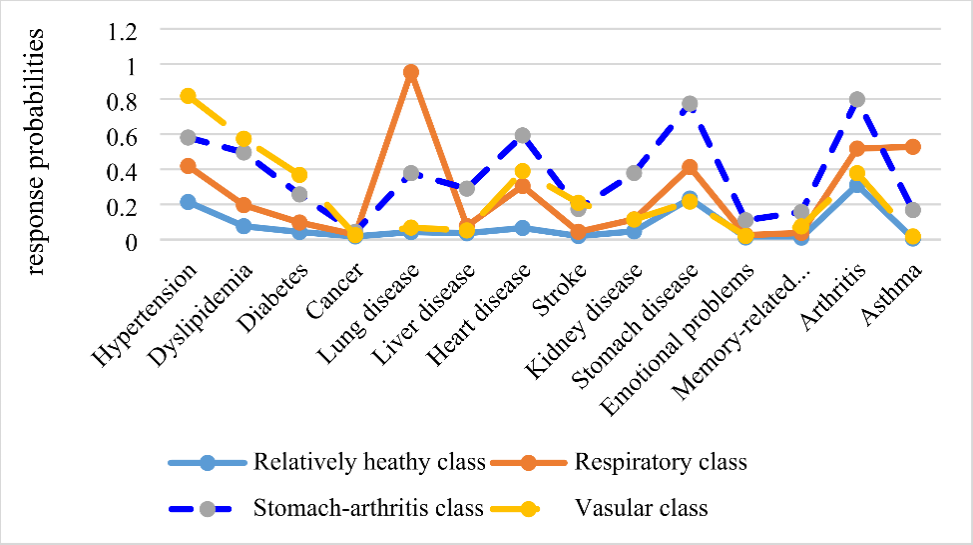
Four-class model of multimorbidity patterns

Of the weighted sample, the prevalence of multimorbidity was 56.73% for all respondents (Table 1). As mentioned above, all respondents were classified into one of the four classes. The *relatively healthy class* consists of respondents with a substantially lower prevalence of all chronic diseases, including 65.02% of the respondents. The *respiratory class* was composed of respondents with a higher prevalence of chronic lung disease and asthma. The *stomach-arthritic class* included respondents with a higher prevalence of stomach/digestive diseases and arthritis. The *vascular class* was composed of respondents with a higher prevalence of hypertension, dyslipidemia, diabetes, heart disease and stroke (See Fig. 2). About 6.39%, 9.99% and 18.60% of the respondents were assigned to the respiratory class, stomach-arthritic class and vascular class, respectively.

### Multimorbidity and health care utilization

In this study, 73.1% of outpatient and 79.2% of inpatient service were consumed by multimorbid patients. Logistic regression analysis was conducted to examine the association between multimorbidity and health care utilization. After controlling for Sociodemographic factors, health behavior and health status, multimorbid participants used more outpatient services (OR = 1.89, 95% CI: 1.65–2.17) and more inpatient services (OR = 2.52, 95% CI: 2.22–2.86) compared to the non-multimorbid participants. Older participants used more inpatient care (OR = 1.02, 95% CI: 1.01–1.02) but less outpatient care (OR = 0.98, 95% CI: 0.98–0.99). Respondents who usually drank, rated their health as good used less health service. And those who had difficulty in ADL used more outpatient (OR = 1.40, 95% CI: 1.24–1.60) and inpatient care service (OR = 1.82, 95% CI: 1.61–2.06). Respondents with health insurance used more health service compared to those without health insurance (Table 2).

**Table 2:** Association between multimorbidity and health care utilization

Variables	Outpatient care utilization		Inpatient care utilization	
	OR (95% CI)		OR (95% CI)	
**Multimorbidity** (ref = no)				
Yes	1.89 (1.65–2.17)***		2.52 (2.22–2.86)***	
**Age**	0.98 (0.98–0.99)***		1.02 (1.01–1.02)***	
**Gender** (ref = Male)				
Female	1.03(0.90–1.24)		0.83 (0.70–0.99)*	
**Marriage status** (ref = Married**)**				
Windowed	1.05 (0.90–1.23)		1.03 (0.88–1.20)	
Others	0.90 (0.63–1.29)		0.77 (0.52–1.14)	
**Education (**ref = Sishu/home school and below**)**				
Elementary school	1.14 (0.99–1.32)		1.09 (0.96–1.25)	
Middle school	0.98(0.82–1.16)		1.00 (0.84–1.19)	
High school and above	1.24 (1.02–1.51)*		0.99 (0.81–1.21)	
**Residence** (ref = Rural)				
Urban	0.94 (0.82–1.07)		1.09 (0.96–1.23)	
**InPCE**	1.11(1.04–1.18) **		0.99 (0.93–1.05)	
**Alcohol consumption (**ref = Never**)**				
Occasionally	1.09 (0.84–1.41)		0.76 (0.61–0.95)*	
Usually	0.79 (0.68–0.92)**		0.58 (0.50–0.67)***	
**Smoking status (**ref **=** Never**)**				
Quit	0.92 (0.61–1.37)		1.01 (0.69–1.48)	
Current	0.95 (0.79–1.15)		1.09 (0.93–1.28)	
**Difficulty in ADL(**ref **=** No**)**				
Yes	1.40 (1.24–1.60)***		1.82 (1.61–2.06)***	
**Self-rated health (**ref = Less than good**)**				
Good	0.55 (0.46–0.67)***		0.45 (0.38–0.54)***	
**Health insurance** (ref = None)				
UEBMI	1.62 (1.16–2.28)**		3.48 (2.38–5.10)***	
URBMI	1.47 (1.07–2.02)*		2.78 (1.91–4.03)***	
NRCMS	1.47 (1.09–1.98)*		2.70 (1.90–3.85)***	
Others	2.20 (1.07–4.52)*		2.25 (1.17–4.35)*	

OR: odds ratio; CI: confidence interval; ADL: Activities in Daily Life * p < 0.05, **p < 0.01, ***p < 0.001

**Fig. 3:**
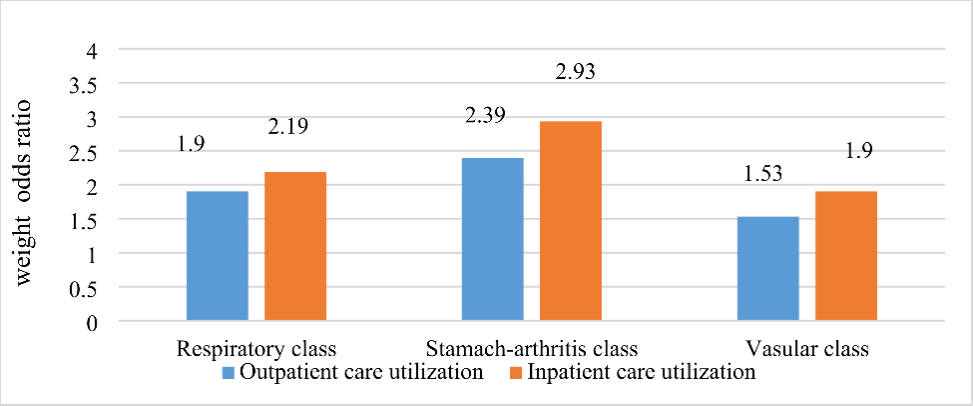
Weighted odds ratio of outpatient and inpatient service utilization by multimorbidity patterns

Another logistic regression was conducted to explore the association between multimorbidity pattern and health care utilization (Covariates include age, gender, marital status, education, residence, InPCE, alcohol consumption, smoking status, difficulty in ADL, self-rated health and health insurance were controlled). The relatively healthy class was employed as the reference group, giving that respondents belonging to this pattern had the lowest probabilities of chronic diseases. The respondents classified into three other patterns used more health service. Compared to relatively healthy class, the respondents classified into respiratory class, stomach-arthritis class and vascular class used more outpatient service (OR = 1.90, 95%CI = 1.57–2.30; OR = 2.39, 95%CI = 2.06–2.78; OR = 1.53, 95%CI = 1.32–1.79 respectively) and more inpatient services (OR = 2.19, 95%CI = 1.83–2.62; OR = 2.93, 95%CI = 2.53–3.40; OR = 1.90, 95%CI = 1.65–2.19 respectively) ( see Fig. 3).

## Discussion

Based on a nationally representative data, the present study investigated the prevalence and patterns of multimorbidity and the association with health care utilization among Chinese middle-aged and older adults. The prevalence of self-reported multimorbidity was 56.73% among middle-aged and older Chinese. Multimorbid respondents used more outpatient and inpatient service. These results are similar to results observed elsewhere [21–23]. Four patterns of multimorbidity: relatively healthy class, respiratory class, stomach-arthritis class and vascular class were identified. Respondents belonging to specific multimorbidity patterns used more health service than their relatively health counterparts.

A growing body of research have examined the prevalence of multimorbidity. The number of chronic conditions varied from two to many, with prevalence from 6.4 to 86.9% in China and from 9 to 83% in South Asia [4]. A study found that persons aged 50 and older among 16 European countries, the prevalence of multimorbidity was 37.3% [13]. These results vary widely, as the heterogeneity was large in terms of definition of multimorbidity, sample and data source among these studies [24]. Several studies also used data from CHARLS, for example, Chen found the prevalence of multimorbidity was 45.5% among Chinese urban residents [11]. Another study found multimorbidity occurred in 42.4% participants aged at least 50 years [4]. The prevalence of multimorbidity in our study was higher than these two studies. A possible explanation is that the follow-up sample of CHARLS became older and there is accumulation of chronic conditions during the aging process [25].

We observed the prevalence of multimorbidity increased with age substantially but slightly decreased among those oldest-old individuals. Possible explanations may be that, first, oldest-old individuals may be a selected sample with fewer chronic conditions. Second, oldest-old individuals may underreport chronic diseases because of recall bias [4]. Female are more vulnerable to multimorbidity compared to male. This gender difference can be explained that female are less likely to underreport their diseases and pay more attention to health status than male [4, 25].

According to the results, multimorbidity was markedly associated with a higher utilization of both outpatient and inpatient care. This result was in line with studies conducted in both developing and developed countries [11, 13, 23, 26, 27]. In a Dutch survey, the mean number of contacts in patients with multimorbidity was significantly higher than patients with one or none chronic diseases (18.3 vs. 11.7 and 6.1 contacts, respectively) [28]. In the Switzerland by Caroline [12], the mean number of consultations per year was 15.7 in the multimorbid compared to 4.4 in the non-multimorbid counterparts. WHO study on global aging and adult health (WHO-SAGE) conducted in India indicated that respondents with three and more chronic diseases were one and a half times more likely to have outpatient and inpatient visit [29]. Multimorbidity requires more diverse and intensive care, which likely explains more health care utilization among individuals with multimorbidity [30].

In the present study, we identified four multimorbidity patterns among middle-aged and older adults in China using LCA. More than 60% of the respondents were classified into the relatively health class, which coincided with previous studies using similar methods, with 60.4% in Korea [31] and 63.8% in Spain [32]. But our result was higher than Zhang et al., who found half of the respondents were classified into the relatively health class because of the older sample in their study. The vascular class was revealed in our study, which consisting of respondents with a high rate of cardiovascular diseases, similar to developed countries [14]. Our study also identified the stomach-arthritic class, similar to previous study [18]. The explanation might be that both digestive diseases and arthritic diseases were of high prevalence among Chinese adults. The other explanation may be that the use of nonsteroidal anti-inflammatory drugs in arthritic patients may cause gastrointestinal ulcers [4].

Belonging to the stomach-arthritis class was strongly associated with higher health service utilization. The prevalence of stomach disease and arthritis is high in middle age and older population in China [33]. Previous study found individuals with arthritis had greater odds of having a hospitalization event and higher number of ambulatory care visits [34]. Another study also found older adults with arthritis were significantly more likely to have a physician visit, hospital admission, outpatient surgery and home health care [35]. The respiratory class also showed a relatively strong association with outpatient and inpatient service utilization. Diseases like lung disease and asthma, which comprise this pattern, are characterised by frequent exacerbations, are most likely responsible for the higher odds of health care utilization [14]. The vascular class showed a higher odds of health utilization. This pattern was characterised by a high prevalence of hypertension, dyslipidemia, heart diseases, diabetes and stroke. This finding was consistent with an Italian study, which was conducted among adults aged 65 years and older, revealed that an increase risk of hospital admissions in adults with heart disease as well as with metabolic patterns [36]. Respondents within this pattern are prone to experience organ decompensations, which may explain higher utilization of health care. However, the associations were even weaker in our study, most likely due to the younger respondents in our study.

Having difficulty in ADL was associated with more health utilization. Which was consisted with previous evidence [37]. Having difficulty in ADL indicated poorer functional state of the respondents and health service consumption increased accordingly. What’s more, respondents had health insurance consumed more health service. Which can be explained by the fact that individuals with no health insurance had limited access to health resources and were liable to be under diagnosis of chronic diseases [38, 39]. Risk factors of multimorbidity and multimorbidity patterns identified in the present study may help develop and implement interventions to prevent more serious consequences of multimorbidity. More attention should be paid to those who were older, having difficulty in ADL and without health insurance.

In this respondents aged 45 years and older in China, 73.1% of outpatient and 79.2% of inpatient services were consumed by multimorbid patients, making this group an important consumer for health care providers. However, multimorbid groups receive little attention in current health service provision in recent China. With the expected rise in multimorbidity in the following decades, this requires more health resources. In case of under treatment for patients with multiple chronic diseases, health systems should be prepared for the future increase in health care utilization.

### Strengths and limitations

A national representative sample of the Chinese middle-aged and older adults were used and the most commonly used definition of multimorbidity were followed in this study [40] to increase the compatibility with previous literature. Though there were some studies on multimorbidity among Chinese adults, these studies focused on the prevalence and patterns [4, 40] of multimorbidity, or only pay attention to urban adults [11].

Limitations of this study include the use of self-report chronic conditions instead of clinical diagnose by healthcare professionals. So the misclassification of diseases or under-diagnosis cannot be ruled out [41]. The CHARLS questionnaire did not ask about all chronic diseases typically included in clinical database studies [42]. Health care utilization was also based on self-report, and therefore may subject to recall bias. Second, a cross- sectional design was used, so the causal relationship between multimorbidity and health care utilization could not be established. But future research needs to investigate such causal relationships by longitudinal data of CHARLS.

### Policy implications

Our findings provide further evidence for targeted interventions and policies to tackle the growing burden of multimorbidity in China. At present, clinical management is largely focused on single diseases, our study support that more focus should be placed on multimorbid individuals, as they need a coordinated, continuous and comprehensive medical care [13]. Owing to population aging and consequent rise in multimorbidity and health service utilization in the future, the management of comorbidities requires a multidisciplinary team to formulate a comprehensive and coordinated care, and through active preventive measures to improve the health of multimorbid patients. Chinese health system should prioritise improving the management of multimorbid patients to increase healthcare efficiency, and improve health outcomes.

## Conclusion

Multimorbidity is highly prevalent among Chinese middle-aged and older adults and is associated with substantially higher health care utilization. Women, old age, having difficulty in ADL, having health insurance were predictors of multimorbidity. Four patterns of multimorbidity: relatively healthy class, respiratory class, stomach-arthritis class and vascular class were identified. Compared to relatively healthy class, the respondents classified into respiratory class, stomach-arthritis class and vascular class used more inpatient and outpatient services. These results may provide insights that could help to manage multimorbidity patients and improve health resource allocation. Strategies to address the growing burden of multimorbidity in China should include effects to improve the management of multimorbid individuals to improve health outcomes associated with aging and reduce associated burden of them.

## Electronic supplementary material

Below is the link to the electronic supplementary material.

Supplementary Material 1

## Data Availability

Please contact CHARLS (China Health and Retirement Longitudinal Study) for data requests. http://charls.pku.edu.cn/zh-CN.

## List of abbreviations

ADL: Activities in Daily Life
CHARLS: China Health and Retirement Longitudinal Study
SRH: Self-rated health
UEBMI: Urban Employee Basic Medical Insurance
URBMI: Urban Resident Basic Medical Insurance
NRCMS: New Rural Cooperative Medical Scheme
