# Supplementary material for: Prevalence, patterns of multimorbidity and associations with health care utilization among middle-aged and older people in China
Source: BMC Public Health. 2023 Mar 21;23:537. doi: 10.1186/s12889-023-15412-5 (PMC10031889; doi:10.1186/s12889-023-15412-5)
Supplement: Supplementary file 1 — Supplementary Material 1 [file 12889_2023_15412_MOESM1_ESM.docx]

**Table 1** Model-fit statistics comparison for latent class analysis

|  | AIC↓ | BIC↓ | aBIC | VLMR-LRT | BLRT | Entropy↑ | proportion |
| --- | --- | --- | --- | --- | --- | --- | --- |
| CLASS1 | 204280.272 | 204390.609 | 204346.118 | — | — | — | — |
| CLASS2 | 194726.761 | 194955.315 | 194863.155 | 0.0000 | 0.0000 | 0.629 | 0.30651/ 0.69349 |
| CLASS3 | 192705.259 | 193052.031 | 192912.201 | 0.0000 | 0.0000 | 0.682 | 0.19311/0.13022/0.67667 |
| **CLASS4** | **191767.533** | **192232.523** | **192045.024** | **0.0039** | **0.0040** | **0.718** | **0.65024/ 0.06386/ 0.09995/ 0.18595** |
| CLASS5 | 190896.725 | 191479.933 | 191244.764 | 0.0000 | 0.0000 | 0.657 | 0.03942/ 0.03794/ 0.55136/ 0.16836/ 0.20292 |
| CLASS6 | 190691.701 | 191393.126 | 191110.289 | 0.0167 | 0.0172 | 0.639 | 0.17051/ 0.16136/ 0.03625/ 0.04775/ 0.03932/ 0.54481 |

AIC Akaike Information Criterion; BIC Bayesian Information Criterion; aBIC Adjusted Bayesian Information Criterion; LMR Lo-Mendell-Rubin Likelihood Ratio Test; BLRT Bootstrap Likelihood Ratio Test
